# Pulmonary hypertension among maintenance hemodialysis patients in Somalia: a hospital-based observational study

**DOI:** 10.1186/s43044-022-00261-1

**Published:** 2022-04-08

**Authors:** Gökhan Alıcı, Mohamud Mire Waberi, Mohamed Abdullahi Mohamud, Ahmed Muhammad Bashir, Ömer Genç

**Affiliations:** 1 Department of Cardiology, Turkey Recep Tayyip Erdogan, Somalia Mogadishu Training and Research Hospital, Mogadishu, Somalia; 2 Department of Cardiology, Agri Training and Research Hospital, Agri, Turkey

**Keywords:** Pulmonary hypertension, Hemodialysis, Somalia, Pericardial effusion, Right atrium

## Abstract

**Background:**

This study aims to examine the prevalence and related factors of pulmonary hypertension (PHT) in patients on hemodialysis (HD) at the only referral institution in Somalia. A total of one hundred and forty-three patients  who had received regular HD therapy for at least three months and underwent transthoracic echocardiography (TTE) were included in the study. Patients with a systolic pulmonary artery pressure (sPAP) value > 35 mmHg at rest on TTE were considered having PHT. The relationship of TTE parameters, demographic, and clinic characteristics of participants with PHT were evaluated.

**Results:**

The number of patients with PHT was 73 (51%). The mean age was 54.2 ± 18.4 years. The majority of patients were 65 years of age or older. (*n*: 46, 32.2%) and 65 (45.5%) were male. Median sPAP was found to be 35 mmHg. Systolic pulmonary artery pressure was positively correlated with right atrium (RA) diameter (*r*: 0.6, *p* < 0.001) and negatively correlated with left ventricular ejection fraction (LVEF) (*r*: − 0.4, *p* < 0.001). In addition, LVEF, RA diameter, presence of pericardial effusion (PE) were found to be independent predictors of PHT.

**Conclusions:**

Pulmonary hypertension has a relatively high prevalence in end-stage renal disease (ESRD) patients on regular HD. Besides, the presence of PE and certain right and left heart parameters were independently associated with PHT.

## Background

Pulmonary hypertension (PHT) is a heterogeneous group of diseases that deteriorates the health-related quality of life and results in poor outcomes if left untreated. Although transthoracic echocardiography (TTE) is helpful in case of clinical suspicion, right heart catheterization is the gold standard diagnostic method. In the 6th World Symposium on PHT, it was recommended to have a mean pulmonary artery pressure of > 20 mmHg at rest for the definition of PHT [1]. Pulmonary hypertension is clinically classified into five groups: Group 1, pulmonary arterial hypertension; Group 2, PHT due to left heart disease; Group 3, PHT due to lung diseases and/or hypoxia; Group 4, chronic thromboembolic pulmonary hypertension (CTEPH); and Group 5 PHT with uncertain mechanisms and/or multifactorial [2]. Additionally, Group 5 consists of individuals with PHT, which may be related to chronic kidney disease (CKD) and other disorders such as chronic hemolytic anemia, sickle cell disease, myeloproliferative disorders, sarcoidosis, and metabolic disorders [3].

The true prevalence of PHT in patients with CKD is poorly known, and its epidemiology has only been defined by limited reports. For instance, in a study on 1876 patients with CKD (stage 1 through 5), right heart catheterization was used for diagnosis, and the prevalence of PHT was found to be 68%, with CKD stage 5 having the highest proportion [4]. Studies have examined the relationship of overvolemia, inflammation, malnutrition, and electrolyte overload with PHT [5–7]. However, the exact mechanisms and risk factors associated with PHT in patients with end-stage renal disease (ESRD) remain unclear.

To our knowledge, data on hemodialysis (HD) patients are lacking in Mogadishu, which has the largest population in the country where the present study was conducted. We are thus of the opinion that it is crucial to provide information about the clinical and demographic characteristics, PHT frequency, and related factors of patients on maintenance HD in Somalia, which has highly limited health opportunities, for evaluating treatment services as well as for improving the effectiveness of treatment and follow-up. For this purpose, we sought to investigate the frequency and possible predictors of PHT in patients with ESRD, thereby closing the gap in question.

## Methods

### Study design and population

This retrospective, observational study was carried out at the Somali-Turkey Training and Research hospital in Mogadishu, the capital of Somalia, where the country's most comprehensive and largest facility is located. A total of 143 consecutive patients aged 14–80 years on regular HD treatment for at least 3 months and applied to the outpatient clinic between January 1, 2021, and April 1, 2021, were included in the study. Patients were excluded if they fulfilled at least one of the following criteria: chronic obstructive pulmonary disease, asthma, acute lung infection, obstructive sleep apnea syndrome, acute/chronic liver failure, previous/acute pulmonary embolism, collagen vascular disease/connective tissue disease, heart failure with reduced ejection fraction, described as left ventricular ejection fraction (LVEF) < 40%, moderate/severe mitral or aortic valve disease (stenosis and/or insufficiency). Patients with a systolic pulmonary artery pressure (sPAP) value > 35 mmHg at rest on TTE were considered having PHT. The study was carried out according to the recommendations set forth by the Declaration of Helsinki on biomedical research involving human subjects. The need for informed consent was waived due to the retrospective nature of the study.

### Transthoracic echocardiography

Echocardiographic examinations were performed by an experienced cardiologist (G.A) who was licensed in Tukey, with Toshiba Aplio ultrasound system (TUS-A500, Shimoishigami, Japan) according to the American Echocardiography guideline [8]. Echocardiographic measurements were gained with all the participants in a left lateral decubitus position and left arm extended over the head. Transthoracic echocardiographic parameters including LVEF, interventricular septum (IVS) thickness, tricuspid regurgitation velocity (TRV), systolic pulmonary artery pressure (sPAP), diastolic dysfunction stages, grade of pericardial effusion (PE), left atrium (LA) diameter, right atrium (RA) diameter were obtained from the registry system for each patient. Pulmonary artery systolic pressure was measured using the Bernoulli equation with TRV and RA pressures. Right atrial pressures were calculated using the size of the inferior vena cava and the degree of collapsibility. Left ventricular ejection fraction was calculated using the Simpson method. Percardial effusion was diagnosed in the presence of an echo-free space between the visceral and the parietal pericardium. The classification was as follows: mild (5–10 mm), moderate/severe (> 10 mm). Left ventricular (LV) diastolic dysfunction was categorized into three stages. Stage I (mild diastolic dysfunction: *E*/*A* < 0.8, deceleration time > 200 ms, average *E*/*e*′ ≤ 8), stage II (moderate diastolic dysfunction or pseudonormal phase: *E*/*A* 0.8–1.5, deceleration time: 160–200 ms, average *E*/*e*′ 9–12), stage III (severe diastolic dysfunction: *E*/*A* ≥ 2, DT < 160 ms, average *E*/*e*′ ≥ 13).

### Blood sample collection and analysis

Venous blood samples were taken from the participants before HD, following an overnight fast of  at least 8 h. All biochemical measurements were made using an automated chemistry analyzer and ready-to-use reagent kits according to standardized protocols provided by manufacturers (Mindray BS 2000 M, China). Measurement of height and weight and calculation of body mass index (BMI) (= kg/m^2^) were made by the HD nurse in the morning (pre-dialysis) on an empty stomach, following standard protocols.

### Statistical analysis

Statistical analyses were performed using IBM SPSS Statistics for Windows version 20.0 (Armonk, NY: IBM Corp.). The normality of continuous variables was assessed by analytical (Kolmogorov–Smirnov test) and visual methods (histograms and probability plots). Continuous variables were expressed as mean ± standard deviation or median (interquartile range), and categorical variables were expressed as number (n) and percentage (%). The Student *t* test and the Mann–Whitney *U* test were used to compare continuous variables. The Chi-square test or the Fisher’s exact test was used for the evaluation of categorical data. All of the significant parameters in the univariate analysis with *p* < 0.05 were selected for the multivariable model, and binary logistic regression analysis was used to determine the independent predictors of PHT. The Hosmer–Lemeshow test was performed to evaluate the goodness of fit of the logistic regression. For each independent variable, the odds ratio (OR) and 95% confidence interval (CI) were determined. A 2-tailed *p*-value of < 0.05 was considered statistically significant.

## Results

**Fig. 1:**
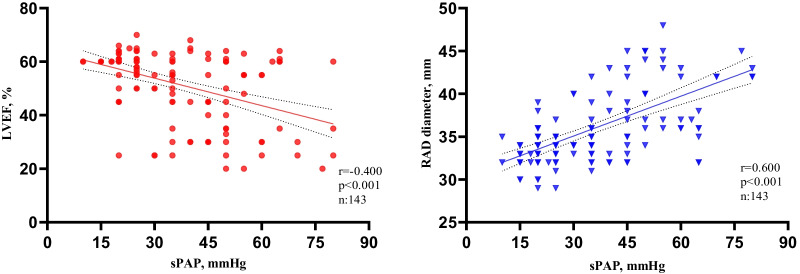
Correlation analysis of sPAP with LVEF and RA diameter. LVEF: left ventricular ejection fraction; RA: right atrium; sPAP: systolic pulmonary artery pressure

A total of 143 patients [65 (45.5%) male, mean age: 54.2 ± 18.4 years] on routine HD for at least three months were included in the study. Among age categories, subjects aged ≥ 65 years were reported to have the highest proportion (32.2%), followed by 55–64 (23.8%) years, 45–54 (19.6%) years, and 35–44 (7.7%) years. Hypertension (*n*: 81, 56.6%) was the most frequent co-morbidity, followed by diabetes mellitus (*n*: 72, 50.3%), whereas smoking (*n*: 5, 3.5%) was the least common. The median sPAP of the study population was found to be 35 mmHg. The number of subjects with PHT was 73 (51%). Most of the TTE parameters were significantly different between the PHT group and the non-PHT group. The PTH group was more likely to have a lower EF than was the non-PTH group (46.5 ± 14.0 vs. 57.5 ± 8.3, *p* < 0.001). Interventricular septum thickness (14.6 ± 3.9 vs. 13.2 ± 2.1, *p* = 0.011), LA (39.4 ± 3.7 vs. 36.8 ± 3.4, *p* < 0.001), and RA diameter (38.2 ± 4.5 vs. 33.6 ± 2.2, *p* < 0.001) were larger in the PHT group than in the non-PHT group. Percardial effusion rate was higher in patients with PHT as compared with the non-PHT group (58.3% vs. 28.6% for mild, 23.6% vs. 2.9% for moderate or severe, *p* < 0.001). Left ventricular diastolic dysfunction was also found significantly more frequently in the PHT group as compared with the non-PHT group (45.2% vs. 31.4% for Grade 1, 35.6% vs. 10.0% for Grade 2 or 3, *p* < 0.001). Laboratory parameters were comparable between the groups, except for the white blood cell (WBC) count which was higher in those without PHT than in those with PHT (6.4 ± 2.6 vs. 7.5 ± 2.8, *p* = 0.014). The detailed demographic, laboratory, and echocardiographic characteristics of the study population according to the presence or absence of PHT are given in Table 1. In the correlation analysis of the relationship between sPAP and various echocardiographic parameters; sPAP was positively correlated with RA diameter (*r*: 0.6, *p* < 0.001) and negatively correlated with LVEF (*r*: − 0.4, *p* < 0.001) (Fig. 1).

**Table 1:** Differences of the baseline parameters between PHT and non-PHT patients

Variables	All	PHT	Non-PHT	p*
	(*n*: 143, 100%)	(*n* = 73, 51%)	(*n* = 70, 49%)	
*Demographic parameters*				
Age (years), mean ± SD	54.2 ± 18.4	55.9 ± 16.2	52.50 ± 20.3	0.260
Male gender, *n* (%)	65 (45.5)	31 (42.5)	34 (48.6)	0.464
Age categories in years, *n* (%)				0.544
14–24	16 (11.2)	5 (6.8)	11 (15.7)	
25–34	8 (5.6)	4 (5.5)	4 (5.7)	
35–44	11 (7.7)	5 (6.8)	6 (8.6)	
45–54	28 (19.6)	17 (23.3)	11 (15.7)	
55–64	34 (23.8)	19 (26.0)	15 (21.4)	
≥ 65	46 (32.2)	23 (31.5)	23 (32.9)	
Hypertension, *n* (%)	81 (56.6)	42 (57.5)	39 (55.7)	0.829
Diabetes mellitus, *n* (%)	72 (50.3)	42 (57.5)	30 (42.9)	0.079
Smoking, *n* (%)	5 (3.5)	3 (4.1)	2 (2.9)	0.684
BMI (km/m^2^), mean ± SD	22.1 ± 2.7	22.3 ± 2.2	21.8 ± 3.1	0.273
*Echocardiographic parameters*				
LVEF (%), mean ± SD	51.9 ± 12.8	46.5 ± 14.0	57.5 ± 8.3	** < 0.001**
TRV(m/s), median (IQR)	2.0 (1.8)	3.8 (0.5)	1.0 (0)	** < 0.001**
sPAP (mmHg), median (IQR)	35 (30)	50 (17.5)	20 (5)	** < 0.001**
IVS (mm), mean ± SD	13.9 ± 3.2	14.6 ± 3.9	13.2 ± 2.1	**0.011**
LA (mm) diameter, mean ± SD	38.2 ± 3.8	39.4 ± 3.7	36.8 ± 3.4	** < 0.001**
RA (mm) diameter, mean ± SD	35.9 ± 4.3	38.2 ± 4.5	33.6 ± 2.2	** < 0.001**
Pericardial effusion, n (%)				** < 0.001**
No effusion	61 (43.0)	13 (18.1)	48 (68.6)	
Mild (5–10 mm)	62 (43.7)	42 (58.3)	20 (28.6)	
Moderate or severe (> 10 mm)	19 (13.4)	17 (23.6)	2 (2.9)	
Diastolic dysfunction, n (%)				** < 0.001**
Normal	55 (38.5)	14 (19.2)	41(58.6)	
Grade 1	55 (38.5)	33 (45.2)	22 (31.4)	
Grade 2 or 3	33 (23.1)	26 (35.6)	7 (10.0)	
*Laboratory parameters*				
Urea (mg/dl), mean ± SD	149.7 ± 49.0	147.1 ± 45.5	152.7 ± 53.0	0.508
NA^+^ (meq/L), mean ± SD	134.9 ± 6.3	135.2 ± 6.2	134.6 ± 6.4	0.600
K^+^ (meq/L), mean ± SD	5.3 ± 0.9	5.4 ± 0.9	5.3 ± 1.1	0.733
Glucose (mg/dl), median (IQR)	115 (56)	107 (48)	121 (63)	0.246
Uric acid (mg/dl), mean ± SD	6.93 ± 1.78	6.77 ± 1.70	7.12 ± 1.88	0.272
Creatinine (mg/dl), mean ± SD	8.9 ± 2.6	8.8 ± 2.3	9.2 ± 2.8	0.316
Hemoglobin (g/dl), mean ± SD	8.4 ± 1.5	8.4 ± 1.6	8.4 ± 1.4	0.958
Platelet count (10^3/uL), mean ± SD	242.0 ± 97.6	242.7 ± 102.5	241.24 ± 92.6	0.932
WBC (10^3/uL), mean ± SD	6.9 ± 2.8	6.4 ± 2.6	7.5 ± 2.8	**0.014**
TSH (uIU/ml), median (IQR)	3.44 (3.4)	3.5 (3.5)	3.2 (3.0)	0.763
Hs-CRP (mg/L), median (IQR)	11.7 (26.3)	8.5 (25.7)	13.6 (28.5)	0.309
ALT (IU/L), median (IQR)	10 (7)	10 (9)	10 (7)	0.984
AST (IU/L), median (IQR)	15 (8)	16 (9)	14 (8)	0.393

ALT: alanine transaminase; AST: aspartate aminotransferase; BMI: body mass index; CRP: C-reactive protein; IVS: interventricular septum; K + : serum potassium concentration; LA: left atrium end-diastolic internal diameter; LVEF: left ventricular ejection fraction; Na + :sodium concentration; PHT: pulmonary hypertension; RA: right atrium end-diastolic internal diameter; sPAP: systolic pulmonary artery pressure; TRV, tricuspid regurgitation velocity; TSH: thyroid stimulating hormone; WBC: white blood cell. *p value < 0.05 was considered significant

On univariate regression analysis, WBC count (OR 0.855, 95% CI 0.753–0.971, *p* = 0.016), LVEF (OR 0.918, 95% CI 0.885–0.953, *p* < 0.001), LA diameter (OR 1.253, 95% CI 1.120–1.403, *p* < 0.001), RA diameter (OR 1.475, 95% CI 1.277–1.704, *p* < 0.001), IVS thickness (OR 1.336, 95% CI 1.080–1.652, *p* = 0.008), LV diastolic dysfunction (Grade 1: OR 4.393, 95% CI 1.951–9.893, *p* < 0.001, Grade 2 or 3: OR 10.878, 95% CI 3.876–30.524, *p* < 0.001), and PE (mild: OR 7.754, 95% CI 3.443–17.462, *p* < 0.001, moderate/severe OR 31.385, 95% CI 6.412–153.618, *p* < 0.001) were all related to higher sPAP levels. After adjustment for the parameters with *p*-value of < 0.05 in univariate regression analysis to determine the predictors of PHT, multivariate logistic regression analysis showed that LVEF (OR 0.945, 95% CI 0.901–0.992, *p* = 0.022), RA diameter (OR 1.384, 95% CI 1.122–1.707, *p* = 0.002), and PE (mild: OR 3.326, 95% CI 1.122–9.053, *p* = 0.019, moderate/severe OR 10.123, 95% CI 1.170–64.966, *p* = 0.010) were independently associated with the presence of PHT (Table 2).

**Table 2:** Univariate and multivariate analyses of predictors for PHT on logistic regression analysis

	OR	95% CI	p^*^	OR	95% CI	p^*^
Age, years	1.011	0.992–1.029	0.258			
Gender, male	0.782	0.404–1.511	0.464			
BMI, kg/m^2^	1.071	0.948–1.211	0.272			
WBC, 10^3/uL	0.855	0.753–0.971	**0.016**	0.836	0.686–1.018	0.074
LVEF, %	0.918	0.885–0.953	**< 0.001**	0.945	0.901–0.992	**0.022**
LA diameter, mm	1.253	1.120–1.403	**< 0.001**	0.864	0.709–1.054	0.151
RA diameter, mm	1.475	1.277–1.704	**< 0.001**	1.384	1.122–1.707	**0.002**
IVS thickness, mm	1.336	1.080–1.652	**0.008**	1.136	0.889–1.450	0.308
*LV diastolic dysfunction*						
Normal		1 (ref)			1 (ref)	
Grade 1	4.393	1.951–9.893	**< 0.001**	1.731	0.478–6.264	0.403
Grade 2 or 3	10.878	3.876–30.524	**< 0.001**	1.576	0.295–8.411	0.594
*Pericardial effusion*						
None		1 (ref)			1 (ref)	
Mild	7.754	3.443–17.462	**< 0.001**	3.326	1.222–9.053	**0.019**
Moderate/severe	31.385	6.412–153.618	**< 0.001**	10.123	1.770–64.966	**0.010**

BMI: body mass index; DD: diastolic dysfunction; IVS: interventricular septum; LA: left atrium end-diastolic internal diameter; LVEF: left ventricular ejection fraction; LV: Left Ventricular; PHT: pulmonary hypertension; RA: right atrium end-diastolic internal diameter; WBC: white blood cell count, − 2Log likelihood: 116,412, Model Chi-square: 80.414, Nagelkerke R^2^ = 0.577, *p value < 0.05 was considered significant, CI: confidence interval, OR: odds ratio

## Discussion

In the present study, we found PHT was prevalent, with a relatively high rate in 73 (51%) subjects among 143 ESRD patients on regular dialysis. Also, LVEF, RA diameter, and the presence of PE were found to be determinants of PHT. This is the first study to investigate the frequency of PHT and parameters independently associated with PHT in patients who received regular HD treatment for at least three months in Somalia.

In meta-analyses conducted on patients who receive regular HD therapy, the prevalence of PHT was reported to be between 23 and 33% [9, 10]. Besides, several small-scale studies have higher rates of PHT, similar to ours [11–13]. Such difference in PHT prevalence among the reports might, in part, be attributed to some advantages stemming from the development level of the country, such as advanced healthcare systems, higher rates of patients' access to effective therapy options, and early diagnosis and treatment. However, there are hardly any studies on PHT in the region. For example, in a recent study in Somalia, we found the prevalence of pulmonary arterial hypertension to be 22.8% [14], while in another study, we reported the prevalence of PHT as 18.2% [15].

There is a wide variety of studies in the existing literature dealing with the relationship between PHT and overall survival in ESRD patients, most of which have shown that PHT is associated with worse poor outcome [16–19]. Furthermore, it has been reported that the association of PHT with mortality remains even after kidney transplantation [20]. Data regarding the prevalence of PHT among ESRD individuals on regular dialysis are also valuable for the preparation of guidelines for the management of complications. Such fragile patients with high diagnostic suspicion on TTE may need to be closely followed up in specialized multidisciplinary PHT units and evaluated for additional tests and specific treatments such as right heart catheterization if necessary [21].

We found that patients with PHT had larger LA, RA, IVS diameters, and lower LVEF and on multivariate regression analysis, LVEF and RA diameter were reported as independent determinants of PHT. Similarly, several previous studies have revealed the link between structural parameters of the heart, such as atrial volume and cardiac chambers, with PHT [22–24].

The relatively high number of patients (49.7%) with PE in the present study suggests that ineffective HD plays a role in the high rate of PHT. The presence of uremic effusion in HD patients requires a multidisciplinary approach, and the dialysis regimen for the individual should be reconsidered when the diagnosis is made. Optimization of water and electrolyte imbalance, acid–base homeostasis by dialysis remains the mainstay of treatment and prevention [25]. The prevalence of uremic effusion in CKD patients is between 2 and 21% [26]. The relationship of PE with PHT and its negative effect on the outcome are well established [27, 28]. Likewise, we found that the presence of PE is an independent predictor of PHT.

## Conclusions

Our findings have shown PHT is a common problem among ESRD patients undergoing regular HD and is independently associated with RA diameter, presence of PE, and LVEF. Due to the high prevalence of PHT in HD patients, it is recommended to screen such patients in order to reduce its effects.

## Limitations

Our study has several limitations. First, our report may be incomplete in reflecting the distribution of PHT of patients on regular HD treatment in the entire Somali population as access to healthcare services in the country is difficult and unequal, and our study was conducted in a single center. Second, right heart catheterization, which is used as the gold standard test in the differential diagnosis of PHT, cannot be performed in the country. Third, although power analysis was performed when planning the study, the sample size is relatively small. Fourth, it should not be ruled out that retrospective studies may have inherent selection bias. Finally, due to the retrospective design of the study, we were unable to present other detailed echocardiographic parameters, especially right ventricular function, and possible confounding factors.

## Data Availability

The analyzed datasets are available from the corresponding author on request.

## Abbreviations

BMI: Body mass index
CKD: Chronic kidney disease
CTEPH: Chronic thromboembolic pulmonary hypertension
ESRD: End-stage renal disease
HD: Hemodialysis
IVS: Interventricular septum
LV: Left ventricular
LVEF: Left ventricular ejection fraction
PHT: Pulmonary hypertension
PE: Pericardial effusion
RA: Right atrium
sPAP: Systolic pulmonary artery pressure
TTE: Transthoracic echocardiography
TRV: Tricuspid regurgitation velocity
WBC: White blood cell
